# Zone adaptive fuel mapping for high resolution wildfire spread forecasting

**DOI:** 10.1038/s41598-025-06402-1

**Published:** 2025-07-01

**Authors:** Paula Sánchez, Irene González, Carlos Carrillo, Ana Cortés, Remo Suppi, Tomàs Margalef

**Affiliations:** Computer Architecture and Operating Systems Department, Universitat Autònoma de Barcelona, 08193 Cerdanyola del Vallès, Spain

**Keywords:** Forest fires, High-resolution fuel mapping, Land cover map, Environmental sciences, Natural hazards, Mathematics and computing

## Abstract

Extreme wildfire events (EWE), although a rare natural hazard, account for a substantial portion of global wildfire damage, requiring proactive anticipation and mitigation due to their increasing occurrence. Wildfire spread simulators are crucial for reducing damage, but they rely heavily on accurate fuel maps, which are often outdated, have low resolution, or are unavailable in many regions. While land cover maps are more up-to-date, high-resolution globally, and widely available, there is no universally accepted method to convert land cover maps into fuel maps. In this work, an automatic methodology for generating high-resolution fuel maps from land cover maps called zone-adaptive fuel mapping (ZAFM) is proposed. ZAFM is a consistent local approach that makes use of public resources to create a fuel map. The proposed methodology has been tested using, as a study case, an EWE that occurred in the north-east of Spain during the summer of 2022. To assess the accuracy of the proposed fuel mapping method, we compared the fire spread forecast using the ZAFM fuel map with fire evolutions based on different fuel maps derived from the land cover map of the study area. The accuracy assessment, based on the *F2-score* metric, reveals that ZAFM achieves the highest *F2-score* of approximately 0.90, while the *F2-scores* for the other fuel maps range from 0.78 to 0.89, with no individual simulation reaching 0.90. ZAFM was also evaluated against other publicly available fuel maps covering Catalonia, and once again achieved higher *F2-scores* in the case study simulations. These results highlight the superior predictive performance of ZAFM and underscore the importance of using up-to-date, high-resolution data to improve wildfire spread forecasts. Furthermore, since ZAFM relies on open-access data maps, it can be applied worldwide with any available high-resolution land cover map.

## Introduction

Extreme wildfire events (EWEs) pose a significant challenge to societies around the world. Although they constitute a minority among all natural hazard events, their destructive power and impact on human lives are often disproportionate^1,2^. These wildfires surpass the current control capacity, even in regions that are well prepared and experienced in firefighting^3–5^. EWEs can be caused by a combination of factors such as dry and hot weather conditions, strong winds, accumulation of flammable materials, and difficult terrain^6,7^. These factors create an environment where wildfires can intensify and spread rapidly, making them difficult to contain and extinguish. Moreover, the increase in global temperature caused by climate change results in a significant increase in the frequency of EWEs^8^. Therefore, preventing and anticipating their behaviour is a critical aspect in minimizing their impact^9,10^. Over the past few decades, significant advances have been performed in forest fire spread simulators with the aim of providing reliable predictions of wildfire behaviour. These tools have been proven to be very useful in providing critical information to incident commanders, assisting in decision-making on evacuation plans, resource deployment, and containment strategies^11–14^.

To accurately assess and respond to a wildfire, it is important to provide information on several key parameters that describe the environment where the fire is taking place. The description of the environment includes data on the landscape, fuel, topography, and weather of the affected area^15^. The main factors driving the creation and evolution of EWEs are weather conditions and fuel characteristics^16^.

In fire simulation, these fuel characteristics are represented through fuel maps, which play a crucial role in fire modelling, analysis and fire risk assessment on a global scale^17,18^. In regions like Australia, integrating fuel models into fire danger rating systems (such as AFDRS) has improved responses to extreme fires^19^. In North America, studies have shown that detailed fuel mapping reduces uncertainty in fire behaviour prediction^20^. Similarly, in South America, research highlights its role in planning controlled burns to prevent uncontrolled wildfires^21^. Therefore, continuing to advance fuel mapping is essential for enhancing fire management strategies worldwide, especially considering that accessing publicly available fuel maps is not always straightforward. Due to the high temporal and spatial variability of fuels, many existing maps can quickly become outdated or may lack the resolution needed to capture local heterogeneity^18^.

In Europe, for example, the availability of public fuel maps for wildland management is particularly challenging, especially at local or regional scale. In some fire-prone areas, such as Spain, Greece, and Portugal, some fuel maps have been developed. For instance, Previncat provides a 20 m resolution fuel map for Catalonia^22^, while ArcFuel delivered forest fuel maps with a 50 m spatial resolution for Greece and Portugal at the national level, as well as for selected regions in Italy (Calabria and Sardinia) and Spain (Málaga and Córdoba)^23^. Additionally, in Portugal, FUMOD has been used to generate fuel maps at a 100 m resolution^24^. However, depending on the region, it is often more difficult to find fuel maps at the local or regional scale. Recently, some European-scale fuel maps have been developed, such as those from FirEUrisk^25–27^ at a 1 km resolution, the EFFIS European Fuel Map at a 250 m resolution^28^, and the FIRE-RES Pan-European Fuel Map at a 100 m resolution^29^. At the continental level, similar efforts have been made in other parts of the world, including South America^30^, Africa^30^, and even at the global scale^31^. At these broader scales, cartography of fuel types has usually been generated from the integration of land use databases^32^ since land cover maps are more easily available and updated periodically, making them a valuable alternative. A land cover map is a map that provides a global classification of Earth’s surface types, categorizing areas into classes such as forests, shrublands, grasslands, croplands, water bodies, urban areas, and more. At the pan-European level, commonly used land cover maps include, for example, the CORINE Land Cover (CLC) map^33^, which categorizes land cover into 44 classes and has been regularly updated since its first release in 1990, with the most recent version from 2018. Another option is the WorldCover map^34^, which provides global land cover products with 11 classes for the years 2020 and 2021 at a 10 m resolution.

Since most forest fire spread simulators do not accept land cover data as fuel input data, a translation procedure to create fuel maps from land cover classes is required. For that reason, scientists typically apply specific heuristics that translate land cover classes into fuel map classifications (commonly known as fuel models) based on their own judgment and the specific characteristics of the burn location. A fuel model defines a set of quantitative vegetation characteristics that can be visually identified in the field.

There are several sets of fuel models, but the most widely used ones are those by Anderson^35^ and Scott and Burgan^20^. The first one is a set of 13 fuel models and the second one expands the fuel classification to around 40 types, therefore, it is difficult to define a direct objective translation of land cover classes into fuel models as there are several models that could be appropriate for a given land cover class. As a result, depending on the criteria employed, the resulting fuel maps could be very different, and consequently the simulations obtained using each fuel map configuration can differ greatly from each other. To address this issue, we propose the *Zone-Adaptive Fuel Mapping* (ZAFM) methodology that provides an objective systematic way to generate a high-resolution fuel map for a given area using open-access data maps.

Other methodologies for generating fuel maps have also been developed. The traditional approach, which relies on fieldwork, is the most widely used but also the most expensive and time-consuming^17^. As a result, efforts are being made to improve vegetation characterization^36–38^ and to develop fuel maps using alternative approaches, such as remote sensing.

Remote sensing has become an increasingly viable alternative due to its affordability and the growing availability of remote sensing products^17^. Several authors have explored fuel type mapping using multispectral and hyperspectral remote sensing data^39^. More recently, many studies have focused on integrating remote sensing with machine learning algorithms to enhance the accuracy and efficiency of fuel mapping. For instance^40–42^, employ neural networks^32^, uses Support Vector Machines (SVM)^43^, utilizes K-Means clustering and Support Vector Machines (SVM), while^44^ applies an ensemble of machine learning methods.

However, all these machine learning-based approaches have certain limitations. They require extensive labelled training data, and the quality and quantity of this data significantly influence the accuracy of the resulting maps^45^. Additionally, models trained in one ecosystem or region often perform poorly when applied to different areas, limiting their effectiveness on a continental or global scale^46^. Training and fine-tuning these models is also resource-intensive^47^. In contrast, the ZAFM methodology does not require training data, is highly generalizable, and can be applied globally wherever low-resolution fuel maps and knowledge about regional fuel types are available. It also has low computational demands, enabling rapid map production. Regarding remote sensing data, using raw imagery typically requires atmospheric correction and extensive pre-processing^43^. Furthermore, data quality can be compromised by atmospheric conditions. In this context, ZAFM presents a clear advantage by relying on an already validated high-resolution land cover map.

LiDAR technology is emerging as an effective alternative for fuel mapping at a regional scale, as it provides valuable information on surface fuels even when obscured by the forest canopy^18,48^. Recent studies have leveraged LiDAR data from UAVs^49^ and Airborne Laser Scanning (ALS)^50^. Additionally, new methodologies that integrate multispectral or hyperspectral data with LiDAR have been developed, offering innovative approaches to wildfire fuel assessment^51–53^.

While LiDAR provides valuable information for fuel mapping, both terrestrial and airborne LiDAR systems are often limited to specific regions and face challenges when mapping at continental and global scales^54^. LiDAR data is costly, and many regions lack access to it. Furthermore, processing high-density point clouds requires significant computational resources and time^55^. In contrast, ZAFM (Zonal Airborne Fuel Mapping) does not require active sensors or expensive data, making it more suitable for large-scale and rapid mapping efforts.

This paper is organized as follows: The description of the ZAFM methodology is presented in the “Methods” section, exemplifying its applicability through a specific case study. In the “Results” section, the metrics used to evaluate the accuracy of the proposal are introduced, followed by the presentation of experimental results. In the “Discussion” section, the results are compared with those from previous studies. Finally, the “Conclusions” section summarizes the key findings and discusses potential directions for future research.

## Methods

As stated in the introduction, there is no standard conversion between the classification of a certain land cover map and the fuel models required to execute any forest fire spread simulator, consequently, scientists apply some heuristics to translate one into the other. Obviously, using heuristics implies that any crosswalk from a land cover map to a fuel map will have an implicit uncertainty due to the decisions made when associating a land cover class with a fuel model.

In order to analyse the impact of using different conversion heuristics on the spread of a wildfire, we used the WorldCover map (WCM)^34^ as the land cover map to be translated, and the Scott and Burgan fire behaviour fuel model classification (FBFM40)^20^ as the set of fuels used to generate the translated fuel map.

At first glance, it is clear that defining a crosswalk from land cover classes to fuel models is not a straightforward assignment. On the one hand, the WCM system primarily focuses on land cover classification based on satellite imagery interpretation. On the other hand, fuel and fire behaviour models consider vegetation characteristics and other combustible materials that influence fire spread and intensity. Although some WCM classes may have a clear correspondence to a fuel model such as artificial surfaces, there exist other classes that are a challenge to classify, like grasslands or forests. This is due to factors such as the complexity and variability of vegetation types, fuel loading, vegetation structure, and spatial arrangement within a given fuel model^56^. Moreover, it is important to note that the transformation between WCM classes and fire behaviour fuel models is context-specific and can vary depending on the region and ecosystem type. Therefore, a single WCM class could correspond to more than one fuel model, and depending on the area, it would be appropriate to choose one or the other. In conclusion, creating a direct class-to-fuel correspondence would require making decisions that introduce subjectivity, and it would not fully represent the local vegetation conditions.

### Study site

**Fig. 1:**
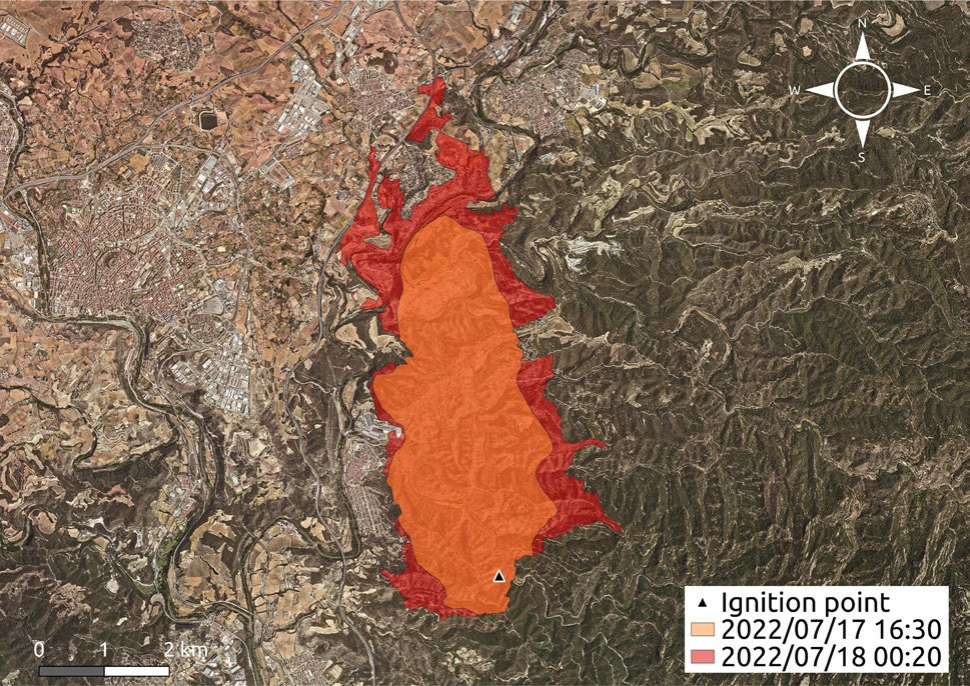
El Pont de Vilomara fire. The triangle marks the ignition point, the orange area shows intermediate spread, and the red area shows the final burned extent^57^. Map derived from the Orthophoto of Catalonia 1:2,500 of the Institut Cartogràfic i Geològic de Catalunya (ICGC), used under a CC BY 4.0 license^58^. Composition created using QGIS v3.34 (https://qgis.org).

To properly analyse the impact of the conversion map process, a real case that can be considered as a near EWE according to the EWE’s characteristics described in^1^ has been studied. The selected case is a forest fire that took place in *El Pont de Vilomara* during the summer of 2022 in the region of Catalonia, Spain^57^. This fire rapidly burned around 14 hectares during the first 15 min and it reached 100 hectares in less than 1 h. Figure 1 shows the point where the fire started (ignition point on July 17 at 13:20), an intermediate burned area (orange shape on July 17 at 16:30), and the final burned area (red shape on July 18 at 00:20). The forest fire spread simulations conducted in this work were initiated using the fire perimeter corresponding to 16:30 hours. This decision was made because the fire’s initial rapid spread, driven by spot fires, resulted in highly unpredictable and intense behaviour. The exact locations of spot fires are difficult to determine and replicate, making it challenging to simulate the fire’s behaviour accurately. To avoid these complexities and focus on the propagation dynamics, all simulations reported in this work use the intermediate perimeter as the initial one.

As it can be observed in Fig. 1, the fire had extreme burning potential on its right flank due to the presence of a large forest mass. Furthermore, it threatened urbanized areas such as *El Pont de Vilomara* village on the left side and a housing estate called *Les Brucades* at the head of the fire front (between the *horns* of the fire).

Since this work aims to highlight how the quality of forest fire spread forecasts is influenced by the fuel map used, this has been the only degree of freedom that has been applied to all the simulations carried out in the experimental section. That is, the rest of the input data required for the forest fire spread simulator, such as topographic parameters (including aspect, elevation, slope, and canopy cover), meteorological conditions, and fuel moisture have been kept constant. The only input data that varies for each simulation is the underlying fuel map.

To fully comprehend the influence of an appropriate choice of heuristic correspondence, we have generated all possible fuel maps by considering all possible class-to-fuel combinations. As the choice of fuel models depends on local conditions, we focused specifically on the fuels relevant to the bioclimatic regime in which the case study fire occurred. Since the fire is located in Catalonia, it falls within the arid/semi-arid bioclimatic regime according to^25^, therefore, and also using the classification proposed in the same work, we included only the fuel models applicable to this regime.

**Fig. 2:**
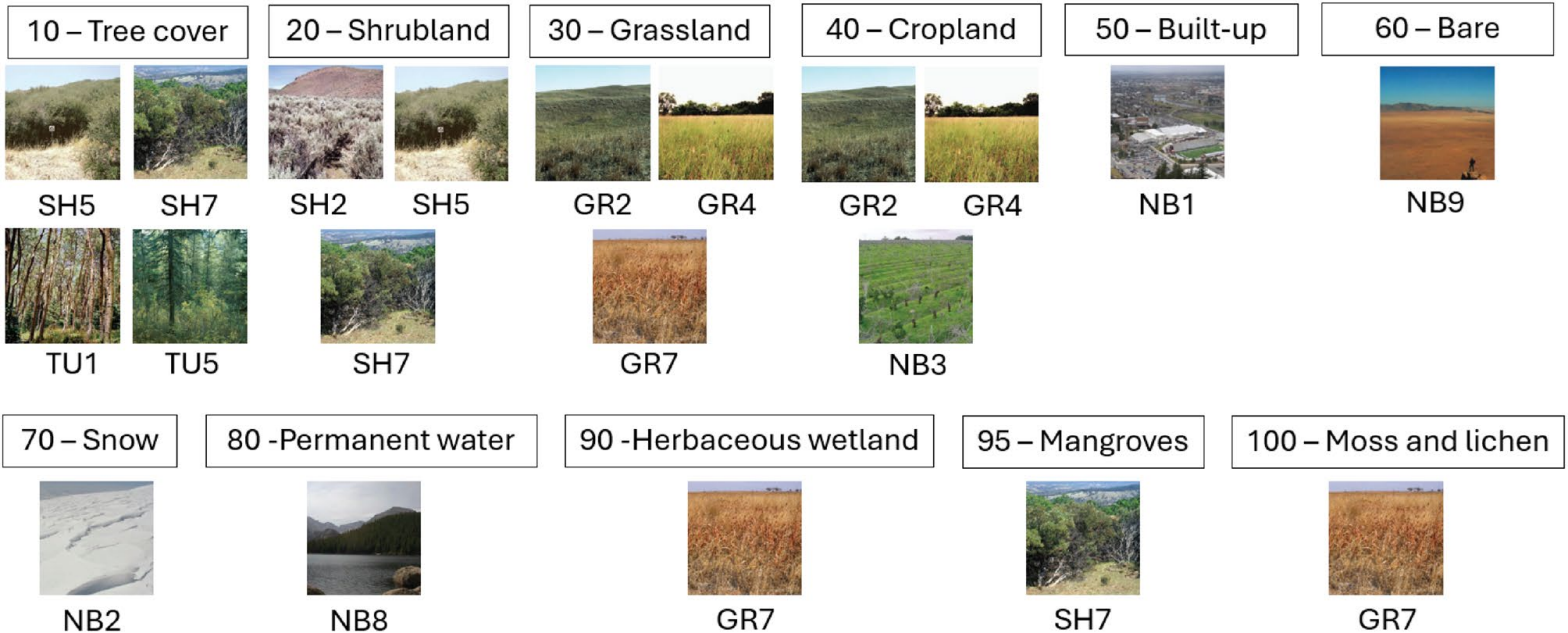
Correspondence groups between WCM classes and FBFM40 fuel models. Only the land cover classes that are present in the study area have been taken into account.

Figure 2 illustrates all possible crosswalks of WCM classes to FBFM40 models for the study area. For each WCM class, a correspondence group can be created, covering all possible FBFM40 models that the WCM class could be substituted for. Therefore, considering all possible combinations of fuel models using these correspondence groups, one comes up with 108 different fuel maps which we will refer to as *scenarios*. Each one of these *scenarios* will be identified with an index *i*, consequently, any particular scenario will be referred to as *scenario i*, where *i* ranges from 0 to 107. At this point, a question arises: which of the possible fuel map scenarios is most suitable for simulating forest fire spread while ensuring maximum accuracy? To answer this question, the next section is devoted to describing a new deterministic methodology for converting a high-resolution land cover map into a unique high-resolution fuel map, based on open data maps. As it will be later shown, this proposal outperforms any heuristic used to translate the land cover map into a fuel map within the study site.

### Zone-adaptive fuel mapping methodology

**Fig. 3:**
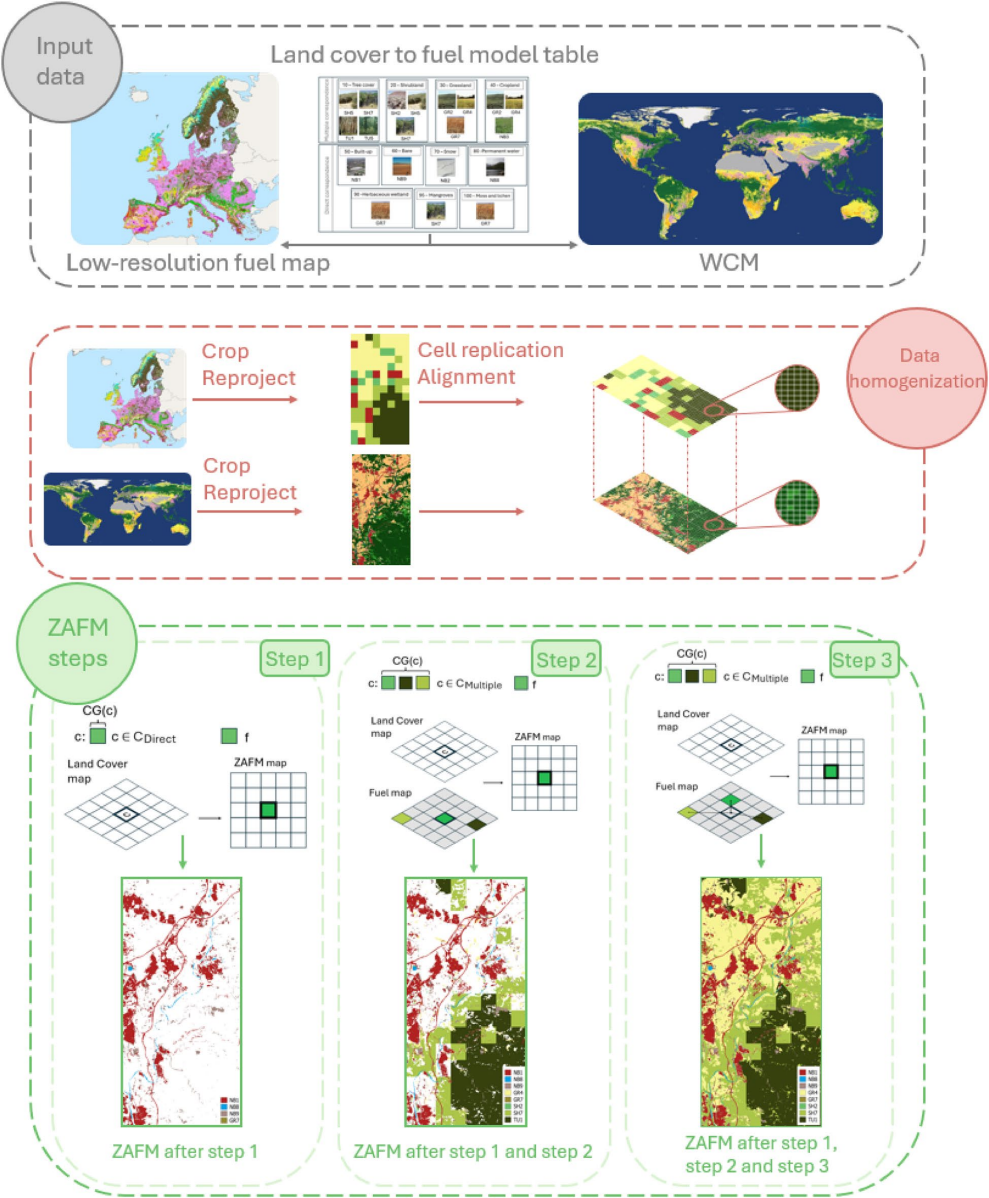
Flowchart of the ZAFM methodology.

As introduced above, to address the challenge of creating a high-resolution fuel map from a high-resolution land cover map, we propose the zone-adaptive fuel mapping (ZAFM) methodology. This approach provides a consistent and deterministic method for generating a high-resolution fuel map for a given area using open-access data. A flowchart illustrating the ZAFM methodology has been included in Fig. 3. As can be observed, ZAFM is composed of three main blocks: Input data, Data homogenization and ZAFM steps. Subsequently, a more detailed explanation of each block is included, as well as the required notation.

#### Input data

To apply this methodology, two maps are required: a high-resolution land cover map of the study area and a low-resolution reference fuel map of the same area to identify the fuel models present in the region (see Input data in Fig. 3). For the case study, *El Pont de Vilomara*, the 10 m WorldCover map from 2021^34^ has been used as a land cover map. Since the fire occurred in 2022, one can consider that this data is sufficiently up-to-date for this study case. As a reference low-resolution fuel map, the European FirEUrisk fuel map^26^ has been used, which is a 1 km resolution map at the European level that employs the FBFM40 fuel models^20^. Since both maps differ in extent and resolution, a data homogenization process must be done before initiating the creation of the high-resolution fuel map (ZAFM scenario). This homogenization process is subsequently described, followed by a detailed description of the ZAFM steps.

#### Data homogenization

The WCM, as its name suggests, is a worldwide map with a resolution of 10 m, while the FirEUrisk fuel map is a pan-European map with a resolution of 1 km. The WCM was downloaded in .TIF format with the projection EPSG:4326, while the FirEUrisk map was also downloaded in .TIFF format but with the EPSG:3035 projection.

Therefore, the first thing to do consists of homogenizing both maps in size, resolution and projection. For this work, we will use the EPSG:25831 projection, so both the WCM and FirEUrisk map will need to be reprojected. This can be done using the following GDAL command:


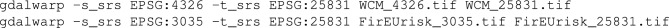


Since the proposed methodology aims to generate a fuel map for the area affected by a fire, both maps need to be clipped to the same extent. This can also be done using a GDAL command. After the -te option, the cropping extent should be defined. xmin = 402812.385, ymin = 4612876.541, xmax = 412046.546, ymax = 4628612.510 define the bounding box coordinates for cropping the map in the Study Site.


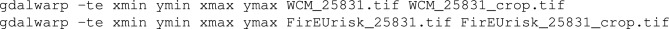


Once both maps cover the same area, they must be adjusted to the same resolution. The main objective of this work is to create a high-resolution fuel map; therefore, the low-resolution FirEurisk map will be corrected to achieve a 10 m resolution. To accomplish this, a cell replication process is applied to the fuel map to match the land cover map’s 10 m resolution:


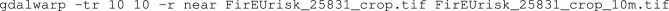


Finally, if both maps are not aligned, we can force them to align using the following command. Here, xmin, ymin, xmax, and ymax represent the coordinates of the corners of $WCM\_25831\_crop.tif$ :


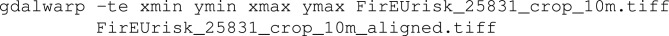


Although this process maintains the original effective resolution, this cell replication process is essential to implement the ZAFM methodology. Data homogenization in Fig. 3 shows the resulting land cover and fuel map after the homogenization process has been applied to the study area.

#### Definitions and notations

Once both maps, the land cover map and the fuel map, are aligned to the same resolution (10 m), the process of generating a high-resolution fuel map can begin. To facilitate the description of the steps involved in this process, certain definitions and notations are required.

The maps involved in this process are rasters that can be represented using a matrix notation. Both the WCM and FirEUrisk map have the same dimensions thanks to the homogenization process, thus they have $n \times m$ cells of a dimension of 10 m × 10 m each, therefore, the resulting ZAFM map will also have the same dimensions. Consequently, one can refer to these three maps as three matrices in the following way:

- $WCM_{n \times m}$: Matrix of size $n \times m$ that represents the high-resolution land cover map.
- $FirEUrisk_{n \times m}$: Matrix of size $n \times m$ that represents the fuel map with augmented resolution to align its cells with the $WCM_{n \times m}$ cells.
- $ZAFM_{n \times m}$: Matrix of size $n \times m$, which corresponds to the resulting map of the proposed methodology.

So any map cell can be referred to as $WCM_{ij}$, $FirEUrisk_{ij}$ and $ZAFM_{ij}$, respectively, where $i = 1, \ldots , n$ and $j = 1, \ldots , m$.

**Fig. 4:**
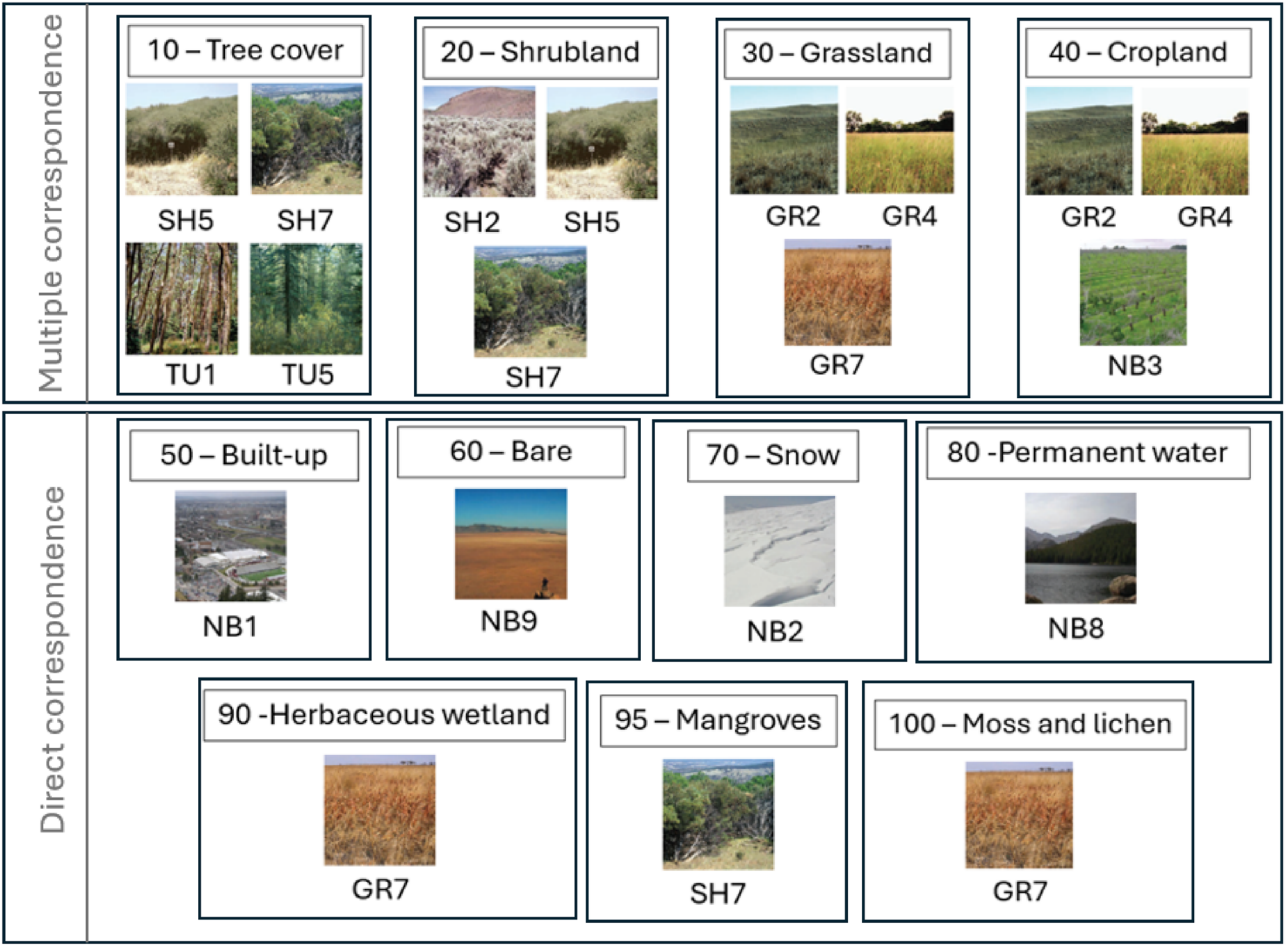
Direct and multiple correspondence groups between WCM classes and FBFM40 fuel models. Only the land cover classes present in the study area have been considered.

Furthermore, we have divided the correspondence groups shown in Fig. 2 into two categories: *Direct* and *Multiple* correspondence groups. The first category includes those in which there is only one FBFM40 fuel model that substitutes the WCM class, such as WCM class 80 (Permanent water), which has a direct correspondence to NB8 fuel model. The set of land cover classes belonging to these *Direct* correspondence group is called $C_{Direct}$. The second type of correspondence groups includes cases where there is more than one option to substitute the corresponding WCM class. For instance, the correspondence group associated with WCM class 20 (Shrubland) is a *Multiple* correspondence group because three fuel models (SH2, SH5, and SH7) can be associated with this land cover class. All land cover classes belonging to this type of correspondence groups are included in the $C_{Multiple}$ set. Figure 4 illustrates this grouping scheme and, for the particular case study reported in this work, the land cover sets $C_{Direct}$ and $C_{Multiple}$ are the following ones:

- $$C_{\text {Direct}} = \{50, 60, 70, 80, 90, 95, 100\}$$
- $$C_{\text {Multiple}} = \{10, 20, 30, 40\}$$

Finally, we have defined a function $CG(c)$ that, given a land cover class $c$, it returns the set of fuel models belonging to the correspondence group of that class. Therefore, for a class belonging to $C_{\text {Direct}}$, $CG(c)$ will return a set, which will contain a single element, meanwhile for a class belonging to $C_{Multiple}$ the obtained set will have more than one element.

#### Global implementation

If one wants to apply the methodology to an area outside of Catalonia, there are different approaches. To apply the methodology to Europe using the FirEUrisk map, it is only necessary to determine the bioclimatic regime^25^ of the area and create the *Direct* and *Multiple* correspondence groups for the corresponding regime (as in Fig. 4). If the methodology is applied in another region of Europe or with a different map instead of FirEUrisk, creating the appropriate *Direct* and *Multiple* correspondence groups using the set of fuel models from the base map to be improved is necessary. If no expert is available to create them, an alternative approach would be to assign all relevant fuel types to each land use category. For example, if the land use category is shrubland, both the Burgan^20^ and Anderson^35^ sets of standard fire behaviour fuel models include the “shrubland” fuel type with multiple possible fuel models. Once the correspondence groups and the map to be improved are available, the methodology can be applied to any region worldwide. If it is not possible to create the *Multiple* correspondence group, an alternative is to improve the map using only direct categories to ensure at least high-resolution representation in areas that are typically non-burnable.

#### ZAFM steps

This section describes the steps involved in the procedure to map a given $ZAFM_{ij}$ cell to a fuel model $f$. The mapping process uses the land cover class $c$ from the corresponding cell of the WorldCover map ($WCM_{ij}$) and the information about fuel models from the FirEUrisk map. This process will be applied to all cells of the *ZAFM* map and consists of three steps, which are the key components of the proposed methodology. For land cover classes belonging to the $C_{\text {Direct}}$ set, this process is straightforward and it is addressed in the first step. However, for those land cover classes belonging to $C_{Multiple}$ the process is more complex and it requires the second and third steps. These steps are applied at each cell $(i,j)$ of the $WCM$ matrix ($WCM_{ij}$) and the corresponding land cover class *c* ($WCM_{ij}$) is used to select which step to apply to map *c* to a fuel model *f* into the corresponding $ZAFM_{ij}$ cell. A detailed explanation of these steps is subsequently introduced.

- *Step 1*:
  If $c \in C_{\text {Direct}}$, $CG(c)$ will be the set of fuels associated with this class, containing a single element, that is, the specific fuel model ($f$) directly associated with $c$.
  Therefore, cell $(i,j)$ in the $ZAFM$ map will be assigned this fuel model, as follows:
  1 $$\begin{aligned} ZAFM_{ij} = f \quad \text {if } c \in C_{\text {Direct}} \text { and } CG(c) = \{f\} \end{aligned}$$
  In other words, this step fills the cells in the $ZAFM$ map where the land cover class of the current cell has a direct correspondence with a specific fuel model. This step is illustrated in Step 1 of the ZAFM flowchart (Fig. 3).
- *Step 2*:
  If $c \in C_{\text {Multiple}}$, $CG(c)$ is a set of potential fuel models for $c$. To identify the specific fuel model ($f$) to assign from the options in $CG(c)$, we consider the fuel model present in the enhanced-resolution fuel map at the same location ($FirEUrisk_{ij}$).
  If $f \in CG(c)$, then the cell $(i,j)$ in the $ZAFM$ map will be assigned this fuel model:
  2 $$\begin{aligned} ZAFM_{ij} = f \quad \text {if } c \in C_{\text {Multiple}} \text { and } f \in CG(c) \end{aligned}$$
  This fuel model assignment is exemplified in Step 2 of the ZAFM flowchart (Fig. 3).
- *Step 3*:
  If $c \in C_{\text {Multiple}}$, $CG(c)$ is a set of potential fuel models for $c$. As in *Step 2*, to identify the specific fuel model ($f$) to assign from the options in $CG(c)$, we consider the fuel model present in the high-resolution fuel map at the same location ($FirEUrisk_{ij}$).
  If $f \notin CG(c)$, a proximity criterion will be used. For that purpose, first, all positions $[k, l]$ in the $FirEUrisk$ map that contains the fuel models within $CG(c)$ are identified. Next, the Euclidean distance from $(i, j)$ to each of these positions is evaluated using the formula:
  3 $$\begin{aligned} d((i, j), (k, l)) = \sqrt{(i - k)^2 + (j - l)^2}. \end{aligned}$$
  The selected fuel model ($f$) from $CG(c)$ will be the one located at the position $(k, l)$ that is closest to $(i, j)$, meaning that the distance $d$ is minimized. Thus, we have:
  4 $$\begin{aligned} ZAFM_{ij} = f \quad \text {if } c \in C_{\text {Multiple}},\ FirEUrisk_{ij} \notin CG(c),\ \text {and } f \in CG(c) \text { at nearest } (k, l) \end{aligned}$$
  This final step is illustrated in Step 3 of Fig. 3.

For an easier understanding of the complete *ZAFM* methodology, a pseudocode of the proposed mapping procedure has been included (Algorithm 1).

**Algorithm 1:**
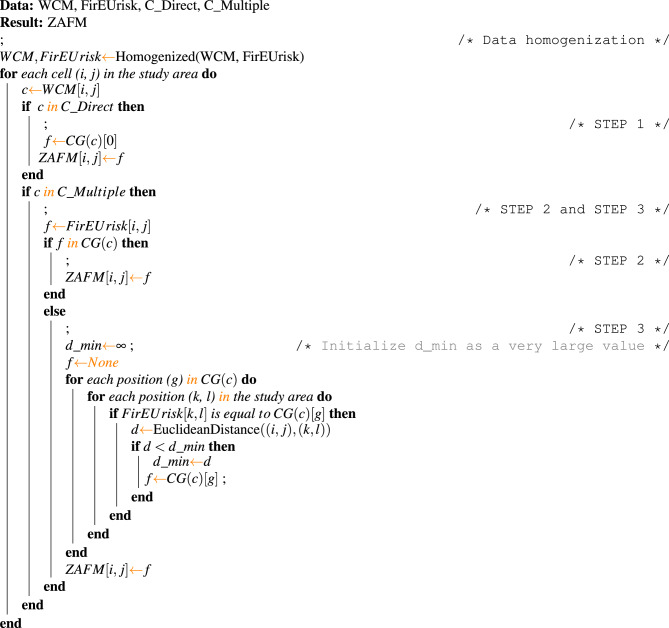
Pseudocode for ZAFM Methodology.

It provides a structured and clear representation of the proposed methodology and serves as a detailed guide for implementing each of the described steps, ensuring reproducibility and transparency of the process. The ZAFM methodology offers several advantages: it is consistent, as it removes subjectivity in assigning fuel models; it is reliable, as it relies on publicly validated maps; and it is deterministic, meaning that given the same high-resolution land cover map and the same low-resolution fuel map, the resulting high-resolution fuel map will always be the same. Additionally, in this method, the assignment of fuel models depends on local conditions, so the same land cover category can be assigned different fuel models based on the specific location of the cell.

## Results

The proposed Zone-Adaptive Fuel Map (ZAFM) methodology defines a deterministic process to convert a high-resolution land cover map into a unique high-resolution fuel map, based on open data sources. The obtained fuel map applying ZAFM is referred to as the ZAFM scenario. This section focuses on evaluating the accuracy of the proposed ZAFM scenario in comparison to other heuristic methods for converting land cover maps into fuel maps. As it was previously mentioned, using the correspondence groups of the study area (Fig. 4), we could generate 108 fuel maps by exploring all logical combination scenarios (from scenario 0 to scenario 107). In order to determine the forecasting agreement between simulation results obtained when using these 108 scenarios plus the ZAFM scenario and the real fire propagation, we have conducted forest fire spread simulations keeping all simulator input data fixed except for the fuel map. As a forest fire spread simulator, we have used *Fire Area Simulator* (FARSITE)^59^. FARSITE is a widely used forest fire spread simulator not only for forecast purposes but also for prevention activities when applying fuel management policies. Despite the results shown in this work correspond to simulations obtained using this forest fire spread simulator, the methodology used is simulator independent, and, therefore, the obtained conclusions can be directly extrapolated to any other wildfire spread simulator.

### Accuracy metrics

Typically, the area burned by a wildfire and the simulated burnt area generated by a certain forest fire spread simulator are represented with shapes on a map (see for example Fig. 1). Since the simulation results often differ from the real behaviour of the forest fire, both shapes (real and simulated) typically mismatch. The location of one shape in the map divides the map into two possible zones: burnt and not-burnt sections. Therefore, forest fire spread prediction can be defined as a categorical forecast, where the observed event is the real area that has been burned by the wildfire and the forecasted event corresponds to the area that the forest fire spread simulator generates as output. It may be noticed that the simulation time must be the same as the real-time of the fire evolution, otherwise, both events could not be compared. Under these conditions, the area where the fire occurs can be divided into four different zones referred to as: *Hits*, *Misses*, *False Alarms* and *Correct Negatives*. *Hits* refer to zones that have been burned in both scenarios (real and simulated), *Misses* are areas burned in the real fire but not in the simulation, *False Alarms* are those areas burned only in the simulation but not in reality and, finally, *Correct Negatives* corresponds to the zone that has not been burnt neither by the real fire nor by the simulation. This last zone (*Correct Negatives*) will depend on the size of the map cut to carry out the simulation, which for the purpose of this study is not relevant, therefore, we do not consider this zone for the metrics subsequently presented.

Based on these concepts, the primary metrics used for accuracy purposes are *False Alarm Rate* (*FAR*) and *Probability of Detection* (*POD*).

*FAR* measures the proportion of the wrong events forecast, see Eq. (5). It is sensitive to *False Alarms* but ignores the *Misses*. Therefore, it could be a good metric to determine whether a simulation generates overestimated areas, that is, the predicted fire evaluations are larger than the real fire propagation area. A perfect comparison has a *FAR* value equal to 0.

5 $$\begin{aligned} FAR=\frac{FA}{(Hits + FA)} \end{aligned}$$

*POD* considers the observed and positively estimated events, see Eq. (6). Thus, it represents the probability of an event being detected. The *POD* is sensitive to *Misses* but ignores *False Alarms*. Consequently, it is a good metric for detecting underestimated forecasts. The ideal value for the *POD* is 1.

6 $$\begin{aligned} POD=\frac{Hits}{Hits+Misses} \end{aligned}$$

Finally, we have also included as an accuracy metric the *F2-score* (*F2*), whose definition is included in Eq. (7):

7 $$\begin{aligned} \textit{F2} = \frac{(1 + 2^2) \cdot \textit{Hits}}{(2^2 \cdot \textit{Hits}) + 4 \cdot \textit{False Alarms} + \textit{Misses}} \end{aligned}$$

The ideal *F2-score* is 1. It prioritizes *Hits* while allowing some *False Alarms* without significant penalties. This is especially important for wildfire simulations, where firefighting efforts are usually not accounted for. In these cases, overestimating the fire’s spread is not overly problematic, as it can represent the fire’s potential behaviour in the absence of intervention.

**Fig. 5:**
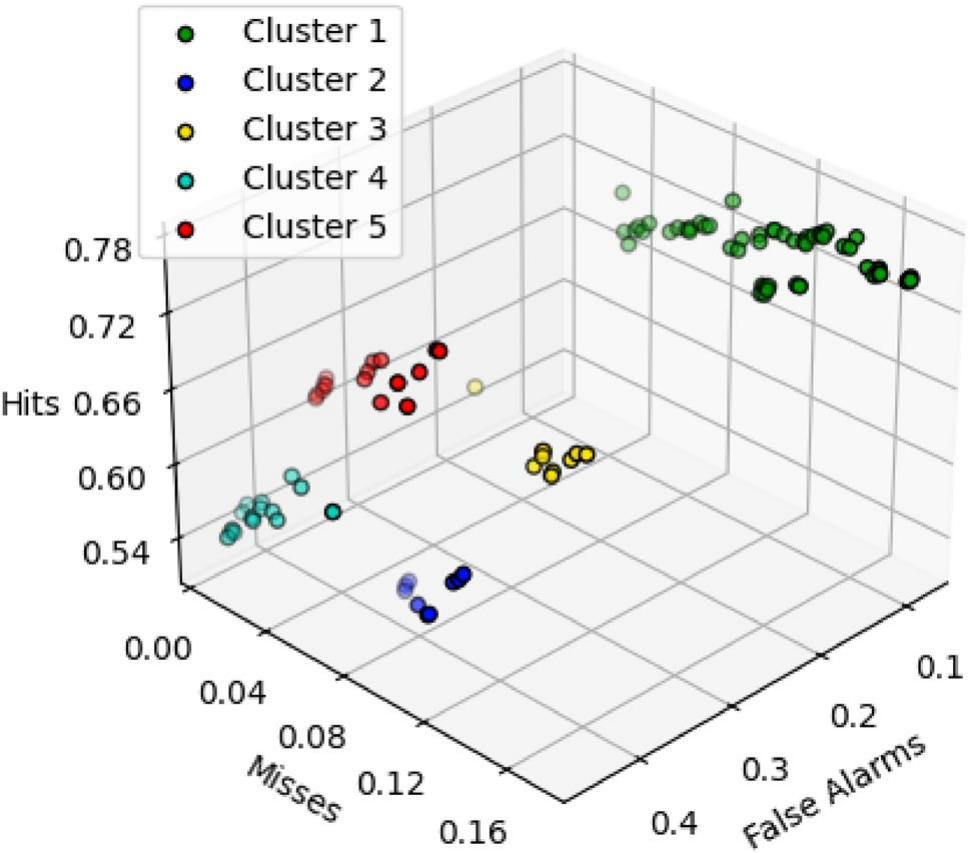
K-means clustering method grouping simulations into five clusters based on the number of *Hits*, *Misses*, and *False Alarms*.

To streamline the graphical presentation of the 109 simulations conducted, 108 using all possible heuristic fuel maps and the one using the proposed ZAFM scenario, while ensuring that all simulations are analysed, we grouped them into 5 clusters. This clustering was based on their similarity in *Hits*, *Misses*, and *False Alarms* relative to the real burned area and was performed using the *K-means* method. The number of clusters for grouping the simulations was determined automatically. Each simulation has been represented as one data point in a three-dimensional space with coordinates corresponding to *Hits*, *Misses*, and *False Alarms*. To ensure fair and consistent comparisons between different fire simulations, these three measures have been normalized. Normalization has been achieved by dividing each measure (*Hits*, *Misses* or *False Alarms*) by the area of the union of the predicted fire and the real fire (*Hits+Misses+FA*). This normalization ensures a clear picture of how well the simulation model performs, regardless of the specific dimensions of the fire. The five clusters are shown in Fig. 5. Each cluster is represented by a different colour, which will be used consistently throughout the rest of the paper for clarity and reference.

#### Accuracy assessment

**Fig. 6:**
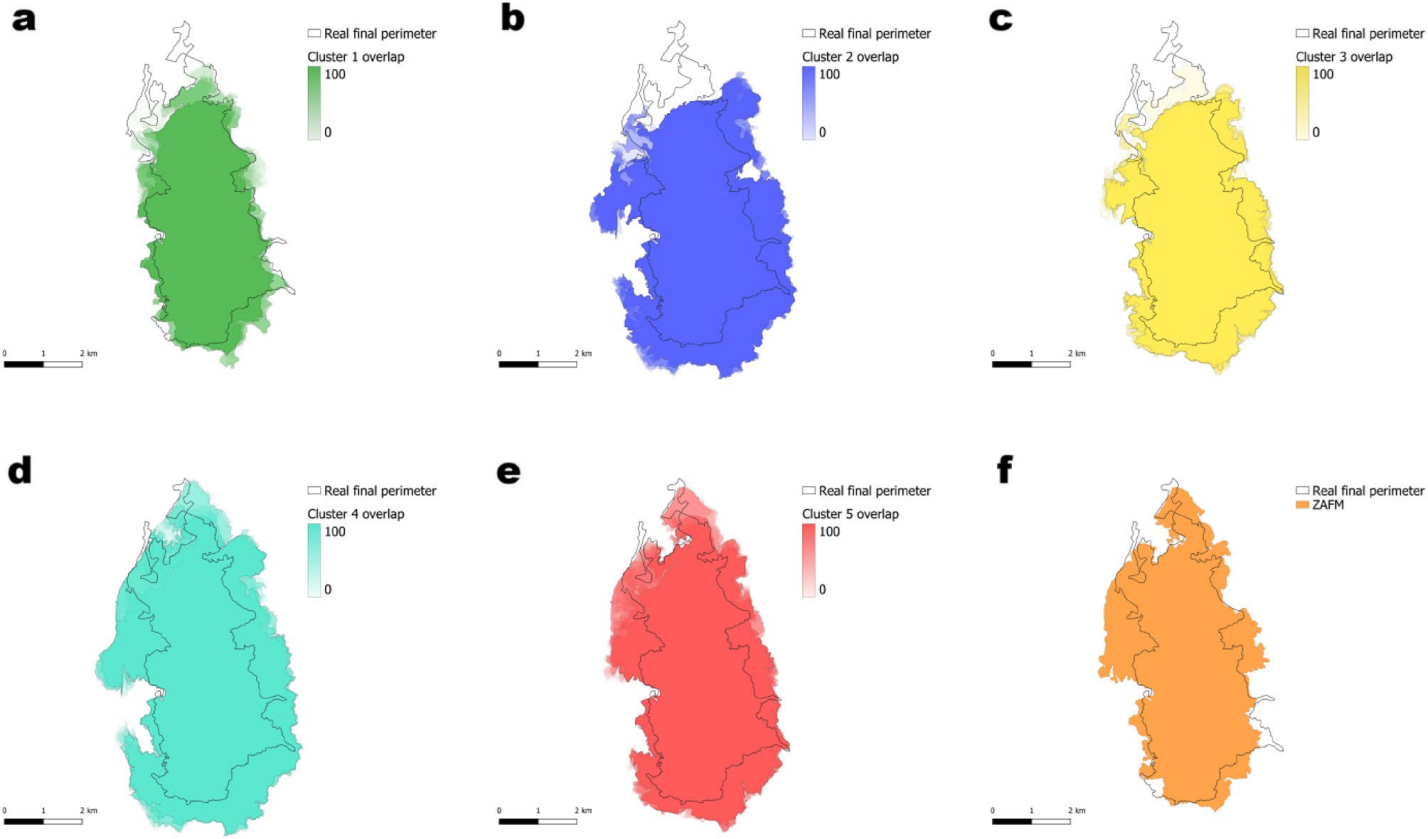
The simulations are shown divided into clusters in Figs. (**a**), (**b**), (**c**) and (**d**). For each cluster, the more colour intensity indicates that more simulations have burned that area (higher overlap). In Fig. (**f**), the simulation obtained using the ZAFM methodology is displayed. The real fire perimeter is outlined in black.

The simulations were conducted using the 109 different fuel maps (108 created using all possible fuel combinations and one generated with the ZAFM methodology), with the topography and weather conditions kept constant, making the fuel map the unique element that varies from one simulation to the other. The simulation duration (determined by the initial and final time of the fire) was 7 h and 50 min. The absence of precise information led to the exclusion of suppression efforts in the fire modelling process. The fire spread shape obtained for each simulation within each cluster and the fire spread shape obtained when using the ZAFM scenario are shown in Fig. 6. In particular, all simulation results obtained for all fuel map configurations belonging to a given cluster have been overlapped in the same image using the reference cluster colour for each overlapped shape. This overlapping process results in a degraded colour image. This coloured map for each cluster, somehow represents a probabilistic map where areas with more intense colours indicate regions with a high probability of being burned, and the transparent areas represent zones with a low probability of being burned. Furthermore, Fig. 6f shows the burnt area obtained when simulating the study case using the ZAFM fuel map. Finally, the real final burned area is the one enclosed by the black perimeter.

If we look at Fig. 6a, we can see that Cluster 1 underestimates the spread of the fire in both the eastern and western regions, while also missing significant parts of the northern area near *Les Brucades*. This could be due to the selected fuel models slowing down the fire’s advance, which has critical implications for early warning systems. An early warning system for forest fires aims to detect and predict the potential severity and spread of a fire as early as possible. The system aims to provide timely information and warnings to enable effective firefighting and evacuation efforts. If the fuel map leads to an underestimation of the fire’s potential severity, it can result in a delayed or inadequate response.

A similar issue is observed in Clusters 2 (Fig. 6b) and 3 (Fig. 6c), particularly in the north, where both clusters also fail to capture the full extent of the fire around *Les Brucades*. However, Cluster 2 tends to overestimate the burned area in both the west, near *El Pont de Vilomara*, and the east, leading to a wider fire spread than seen in reality. Cluster 3, while being slightly better at representing the burned areas, also shares the same tendency to overestimate certain sections.

On the other hand, Clusters 4 (Fig. 6d) and 5 (Fig. 6e) provide a better representation of the burned area, with few *Misses* but a higher number of *False Alarms*. Cluster 4 significantly overestimates the fire’s spread, predicting more extensive burning than what actually occurred. In contrast, Cluster 5 appears to offer the most accurate representation of reality among all the clusters.

When we look at the ZAFM simulation (Fig. 6f), it shows a much closer alignment with the real fire perimeter, effectively balancing overestimation and underestimation, both of which are relatively small. At first glance, it appears to be one of the best representations, however, to properly analyse this aspect, we used the quantitative metrics *POD*, *FAR*, and *F2-score*, which offer a more detailed evaluation of the simulation’s performance compared to what can be observed in the simulations.

Since this study consists of analysing the global accuracy for all scenarios compared to the real fire evolution, all these metrics have been evaluated for all scenarios using the real fire spread as the element to compare to.

**Fig. 7:**
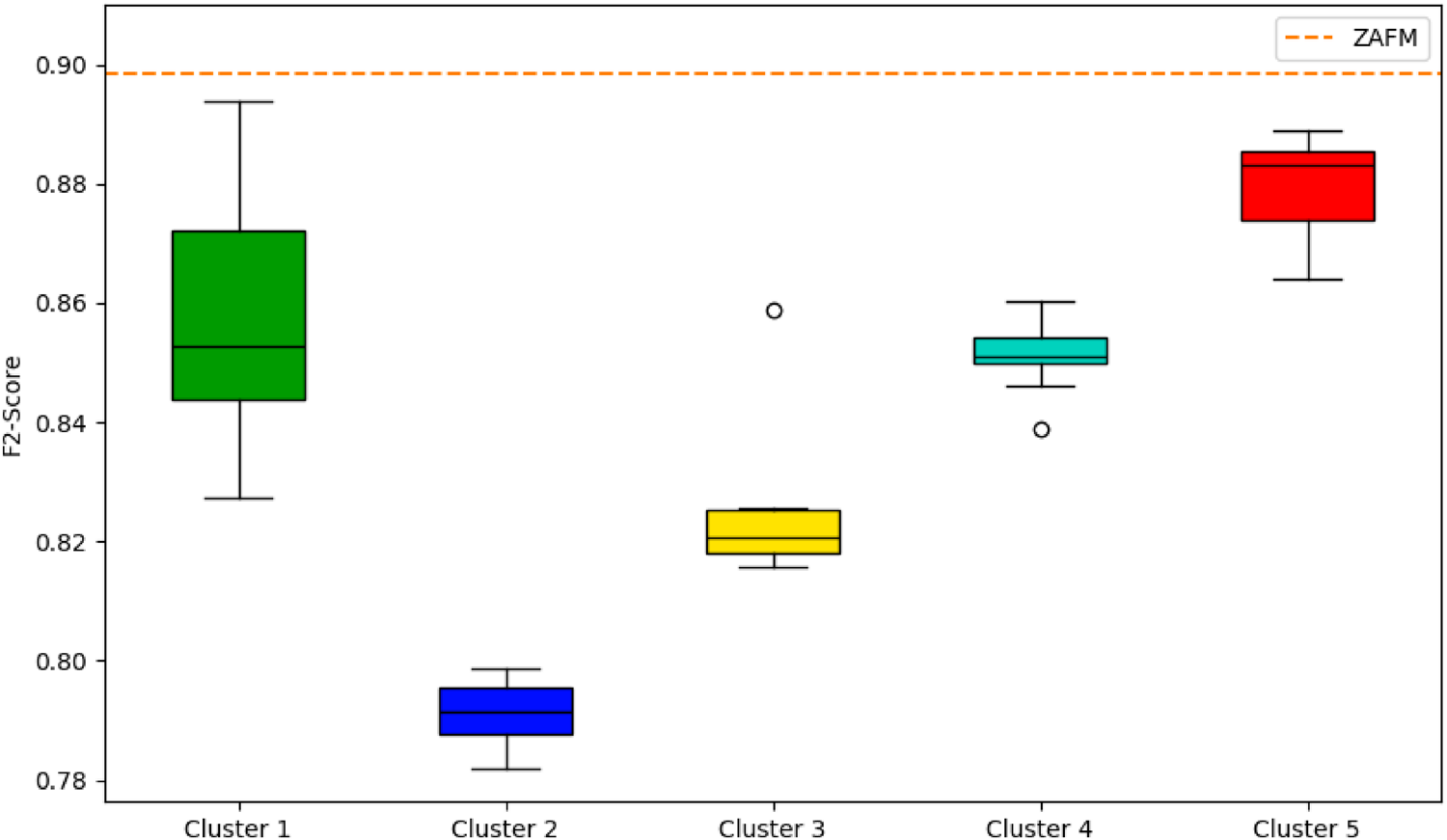
Boxplot of F2-score for the clusters and for the ZAFM (orange dashed line).

To evaluate the performance of the different scenarios and the ZAFM methodology, we created a boxplot displaying the *F2-score* for the five clusters and the ZAFM scenario (represented by the orange dashed line), as shown in Fig. 7. The *F2-score* is the chosen metric for determining which simulation performs best, as it prioritizes the detection of burned areas while accounting for overestimation.

In Fig. 7 we can see that the ZAFM methodology achieves the highest *F2-score*, around 0.90, consistently outperforming all clusters. This indicates that ZAFM methodology is highly effective in predicting the burned areas, achieving a good balance between high detection rates and low overestimation. Cluster 5 shows the best performance among the clusters, with *F2-scores* mostly above 0.86 and the highest median score. This cluster’s performance is closest to ZAFM, suggesting that the scenarios in Cluster 5 are nearly as effective as ZAFM. Cluster 2 has the lowest *F2-scores*, with values clustering around 0.79 to 0.80. This suggests that the scenarios in Cluster 2 are less effective in predicting the burned areas, either due to underestimating the burned extent or overestimating non-burned areas.

Overall, the ZAFM method clearly outperforms the cluster groups, demonstrating its strength in creating high-resolution fuel maps that accurately predict fire spread. Clusters 5 and 1 are the closest in performance to ZAFM, but there is still a noticeable gap, highlighting ZAFM’s superior predictive capability.

## Discussion

As just shown, the ZAFM methodology generates a fuel map that, for the case study, provides the best simulation of wildfire spread compared to 108 other simulations obtained from heuristically generated fuel maps. These maps highlight the complexity of the problem associated with creating accurate fuel maps, allowing for a fair comparison between fuel maps of the same resolution. However, it is possible that none of these maps directly corresponds to an existing fuel map. For this reason, this section compares the simulation results obtained by applying the ZAFM methodology with two existing fuel maps of the study site. The selected methodologies were those for which publicly accessible fuel maps were available for download. The maps used include:

- The PREVINCAT fuel map with a 20m resolution for Catalonia^22^.
- The FIRE-RES Pan-European fuel map with a 100m resolution^29^.

The PREVINCAT fuel map was developed using LiDAR data to extract structural variables from the forest canopy, such as tree height, diameter, canopy cover, and basal area. Additionally, it incorporates existing information on vegetation formations across Catalonia^22^. The Pan-European fuel map FIRE-RES was generated using CORINE Land Cover 2018, ESA WorldCover 2021, the Aridity Index (AI) and a decision tree classification approach^29^. Another map that also covers Catalonia is the EFFIS European fuel map, which has a 250 m resolution^28^. However, this map could not be used because it contains areas with missing data within the study area, making it impossible to perform simulations.

**Fig. 8:**
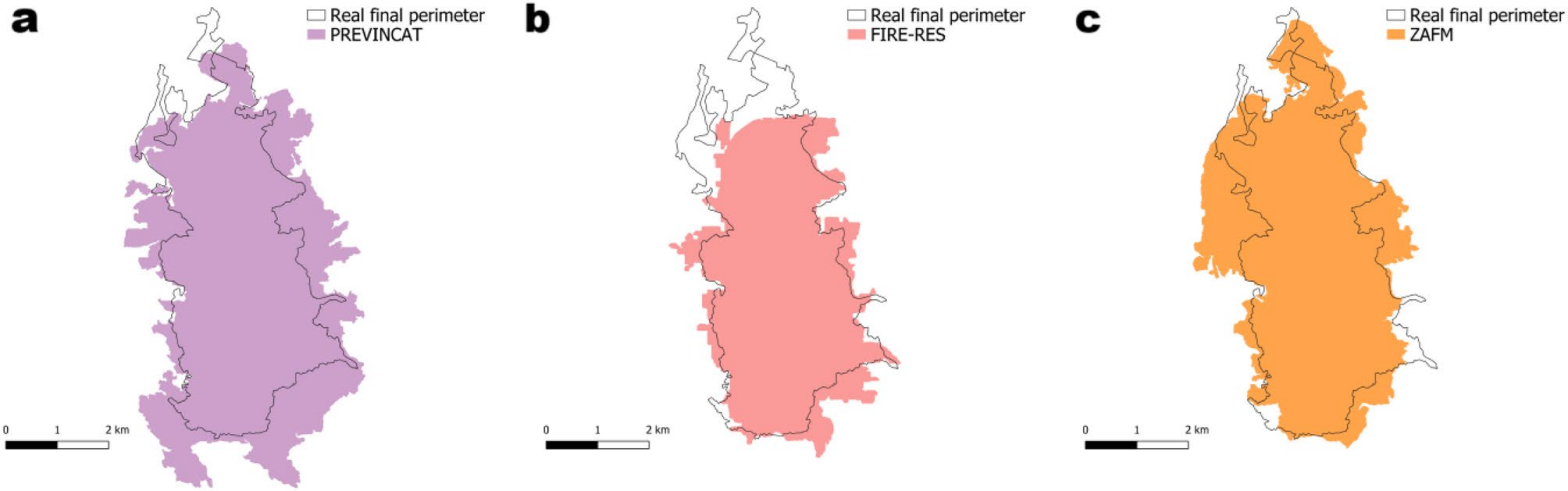
Simulations obtained using (**a**) PREVINCAT fuel map, (**b**) FIRE-RES fuel map and (**c**) ZAFM methodology.

Since the objective was to evaluate the performance of each fuel map, the simulations were carried out using the same input data as the one used for ZAFM. The only variable input in the simulations was the underlying fuel map. To enable simulations, each fuel map was reprojected to EPSG:25831 and cropped to match the extent of the study area. The simulation results have been included and can be seen in Fig. 8.

To make the comparison, the *F2-score* metric was used. Comparison of *F2-scores* on the three fuel maps reveals notable differences in simulation performance. The simulation based on the ZAFM methodology achieved the highest *F2-score* (0.90), followed by PREVINCAT (0.86), and finally FIRE-RES (0.84).

The PREVINCAT fuel map integrates high-resolution LiDAR-derived forest structure variables (such as height, diameter, canopy cover and basal area) together with detailed field-based vegetation formation data. This combination typically provides a very accurate representation of fuel characteristics. Consequently, the results using this map should initially be optimal. However, ZAFM outperforms PREVINCAT. The main reason for this result is temporal factors. PREVINCAT is based on data from 2016, generating an updated PREVINCAT map involves a lot of field work and manual actions that require a long period of time. By contrast, ZAFM uses more recent satellite-derived information from 2021, which is closer in time to the fire event.

In contrast, the FIRE-RES fuel map achieved the lowest *F2-score*. Although FIRE-RES and ZAFM share some input data (ESA WorldCover 2021) the lower resolution of FIRE-RES likely limits its ability to capture fine-scale fuel heterogeneity, which can lead to a slight reduction in accuracy compared to the other maps.

Overall, these results suggest that while detailed field and LiDAR-based maps like PREVINCAT are highly valuable, up-to-date information and finer resolution (as used in ZAFM) may play a decisive role in improving the predictive accuracy of wildfire simulations.

## Conclusions

Extreme wildfire events (EWEs) are becoming one of the most serious natural hazards due to climate change. Dealing with this issue requires informed decisions based on a solid understanding of fire behaviour, for which fire spread simulators are essential. However, the accuracy of these simulations relies heavily on the quality of fuel maps, which are difficult to obtain, especially when high-resolution, up-to-date, and detailed maps are needed. To address this, we proposed the Zone-Adaptive Fuel Mapping (ZAFM) methodology, which offers a novel, consistent, and deterministic approach for converting high-resolution land cover maps into high-resolution fuel maps. ZAFM uses public resources and it adapts to local conditions, providing a more accurate picture of vegetation and fire behaviour. The proposal ZAFM methodology has been applied to a case study, *El Pont de Vilomara* wildfire that took place in 2022 in Catalonia, Spain. To carry out the experimental study, the FARSITE forest fire spread simulator was used. The evolution of the *El Pont de Vilomara* fire has been simulated using 109 fuel map scenarios, 108 heuristic fuel maps derived by exploring all possible combinations of land cover categories matched to fuel models and the ZAFM scenario. The obtained burned areas have been compared to determine the accuracy of the proposal. The results show that ZAFM consistently outperforms all other fuel maps, achieving the highest value for the *F2-score* metric (approximately 0.90), which indicates a superior balance between accurately detecting burned areas and minimizing overestimation.

ZAFM was also compared with other publicly available fuel maps that cover Catalonia, and simulations were conducted for the case study to assess its performance against other methodologies. The results showed that ZAFM consistently outperformed the two other maps used in the comparison, suggesting that up-to-date information and higher resolution play a critical role in enhancing the predictive accuracy of wildfire simulations.

By relying on a consistent and zone-adaptive approach, ZAFM eliminates the subjectivity involved in the manual conversion of land cover data to fuel maps. This methodology provides a more reliable alternative for wildfire simulations and has the potential to be applied in various regions, contributing to more informed and effective fire mitigation strategies.

## Data Availability

The data that support the findings of this study are available from the corresponding author PS on request.
